# Adapting operational research training to the Rwandan context: the Intermediate Operational Research Training programme

**DOI:** 10.1080/16549716.2017.1386930

**Published:** 2017-11-09

**Authors:** Jackline Odhiambo, Cheryl L. Amoroso, Peter Barebwanuwe, Christine Warugaba, Bethany L. Hedt-Gauthier

**Affiliations:** ^a^ Research Department, Partners In Health/Inshuti Mu Buzima, Kigali, Rwanda; ^b^ Department of Global Health and Social Medicine, Harvard Medical School, Boston, MA, USA

**Keywords:** Research capacity building, mentorship, training, learning by doing, Africa, health research, SORT IT

## Abstract

**Background**: Promoting national health research agendas in low- and middle-income countries (LMICs) requires adequate numbers of individuals with skills to initiate and conduct research. Recently, non-governmental organizations (NGOs) have joined research capacity building efforts to increase research leadership by LMIC nationals. Partners In Health, an international NGO operating in Rwanda, implemented its first Intermediate Operational Research Training (IORT) course to cultivate Rwandan research talent and generate evidence to improve health care delivery.

**Objective**: This paper describes the implementation of IORT to share experiences with other organizations interested in developing similar training programmes.

**Methods**: The Intermediate Operational Research Training utilized a deliverable-driven training model, using learning-by-doing pedagogy with intensive hands-on mentorship to build research skills from protocol development to scientific publication. The course had short (two-day) but frequent training sessions (seven sessions over eight months). Trainees were clinical and programme staff working at the district level who were paired to jointly lead a research project.

**Results**: Of 10 trainees admitted to the course from a pool of 24 applicants, nine trainees completed the course with five research projects published in peer-reviewed journals. Strengths of the course included supportive national and institutional research capacity guidelines, building from a successful training model, and trainee commitment. Challenges included delays in ethical review, high mentorship workload of up to 250 hours of practicum mentorship, lack of access to literature in subscription journals and high costs of open access publication.

**Conclusions**: The IORT course was an effective way to support the district-based government and NGO staff in gaining research skills, as well as answering research questions relevant to health service delivery at district hospitals. Other NGOs should build on successful programmes while adapting course elements to address context-specific challenges. Mentorship for LMIC trainees is critical for effectiveness of research capacity building initiatives.

## Background

Despite recent calls by the World Health Organization (WHO) to strengthen health research capacity in low- and middle-income countries (LMICs) [1–3], building and sustaining health research capacity has remained a challenge [4–7]. Reviews of authorship have found LMIC authors under-represented on scientific papers in general as well as on papers about LMICs [8–10]. Factors such as insufficient research infrastructure, domestic and international research funding shortages, few trained researchers at the university level, and inadequate engagement of local researchers contribute to low research capacity in LMICs [4–7]. To address these gaps, non-governmental organizations (NGOs) have joined research capacity building efforts in recent years, providing training and mentorship to individuals who cannot access academic training due to the remoteness of their workplace or lack of time and resources [11,12].

The Structured Operational Research and Training Initiative (SORT IT) [13], developed in 2012 by the Special Program for Research and Training in Tropical Diseases hosted at the World Health Organization (WHO-TDR), is an example of a successful and well-documented capacity building initiative that involves collaborative partnerships [12,14]. Part of SORT IT’s training and capacity building activities were adapted from the operational research training model initially conceived by Medecins Sans Frontieres-Luxembourg and the International Union Against Tuberculosis and Lung Disease (the MSF-Union model of training) [14]. At its core, SORT IT provides a three-week training on protocol development, data analysis and paper writing, using a deliverable-driven and learning-by-doing pedagogy [15,16] with hands-on mentorship [17–20]. By March 2016, SORT IT had conducted 38 courses in Africa, Asia, Europe, Latin America and the South Pacific for 428 participants from 82 countries. These courses resulted in 315 papers submitted to peer-reviewed journals, of which 265 (84%) are published; sixty-five percent of publications from the first 14 courses have led to changes in policy or practice, including revisions of national guidelines and policies at national, sub-national, hospital and NGO levels [18,19].

Recognizing that there is ‘more than one way to slice the cake’ [20], the SORT IT initiative is encouraging different programmes to adapt the training model to meet the needs and constraints of specific contexts. To that end, Partners In Health/Rwanda (PIH/Rwanda), an international NGO supporting the Rwanda Ministry of Health (RMoH) in health service delivery, training and research in three rural district hospitals, developed an Intermediate Operational Research Training (IORT) course in 2013. The team based IORT on the SORT IT model but made several modifications to suit the Rwandan context. Below, we describe the implementation of the first PIH/Rwanda IORT, noting the training goals or contextual challenges that motivated our decisions to adapt the SORT IT model. These details can support other programmes developing or adapting research training models.

## Implementation of Intermediate Operational Research Training

### Overall course goals

The Rwanda Ministry of Health (RMoH) has prioritized health research and requires that every research activity includes capacity building for Rwandans [21–24]. The RMoH requires its employees to participate in research to promote the use of evidence in practice and policy-making and foster an active research environment [25]. In response, the PIH/Rwanda Research Department developed a three-level approach to research capacity building in 2012. Our goals included fostering interest in research through an Introduction to Research Course [26], building research skills through IORT, and facilitating research leadership through scholarships for advanced research degrees. These efforts aimed to cultivate Rwandan research talent and produce research evidence to improve health delivery.

To build research skills at the district level, the IORT focused on operational research, defined as ‘the search for knowledge on interventions, strategies or tools that can enhance the quality, effectiveness or coverage of programs in which research is conducted’ [12]. Operational research generates evidence that is immediately relevant to local health delivery and has potential for improving practice [27]. We adopted an outputs-oriented model with defined and time-specified milestones, using a learning-by-doing approach to allow trainees to apply concepts to projects in real time while receiving hands-on intensive mentorship [17,20].

### Course frequency and content

The majority of clinical and programme health workers in Rwanda, especially in the rural settings, have limited research exposure. Additionally, the shortage of health care workers in the country leads to high workloads [21] that make it difficult for clinical trainees to leave work for long periods of time to participate in research training. To address both challenges, we opted for short but frequent training sessions, with two training days for each of seven sessions conducted every four to six weeks over eight months (Table 1). The more frequent sessions allowed gradual development of research projects with more regular contact between trainees and mentors and reduced the amount of time trainees spent away from work. All training participants and mentors lived in Rwanda within a 2.5-hour drive of the training location in Kigali, making frequent course meetings feasible.

**Table 1.** Description of the PIH/Rwanda’s IORT course, with adaptations to SORT IT course highlighted.

Area	SORT IT Course	IORT Course	Rationale for differences/adaptions
Approach	Outputs-oriented model through learning-by-doing approach	Outputs-oriented model through learning-by doing-approach	
Frequency/length of training	3 6-day modules, with Module 1 and Module 2 back-to-back and Module 3 8 months later. Duration of 9–10 months.	7 2-day modules, every 4–6 weeks. Duration of 8 months.	Because of a nascent research culture in Rwanda, with the majority of trainees having limited research exposure, a stepwise development of the projects with more regular contact with course mentors was necessary to make research accessible and to strengthen learning outcomes.
Milestones	4 milestones, all completed by a deadline to stay in the programme and final manuscript submitted to a peer-reviewed journal to receive certificate of completion.	7 milestones, all completed to stay in the programme and to receive certificate of completion. The first 6 milestones completed prior to the next training session. The final manuscript must be submitted to a peer-reviewed journal but had no specific deadline.	
Training format	Lectures, break-out writing sessions with mentorship, plenary sessions for group feedback and a practicum period to implement skills.	Lectures, break-out writing sessions with mentorship, plenary sessions for group feedback and a practicum period to implement skills.	
Target trainees	Programme and clinical staff.	Programme and clinical staff.	
Trainee selection and number	Individually selected, based on strength of application and feasibility of proposal. 12 trainees selected with 12 research projects.	Applied and selected in PIH-RMoH pairs based on strength of the application and strategic value of the research and of training the applicants. 5 pairs selected with 5 projects.	We trained in pairs to train more individuals given limited funding for projects and few mentors available to provide needed support.
Facilitation and Mentorship	8 mentors for modules 1 and 3, a pair of mentors (senior and junior) with 3 mentees. 4–6 mentors for Module 2, each with 2–3 mentees. In-person mentorship offering during training and long-distance mentorship during practicums.	2 mentors with 1 research fellow. The 2 mentors provided support for the 5 teams for modules 1–6, for module 7, each mentor paired with 2–3 teams to promote intensive support. In-person mentorship offering during training and practicums.	The lower mentor-to-trainee ratio reflected available resources. Due to high clinical and programmatic workload for trainees, mentors travelled to trainee work place to provide in-person mentorship during practicums.
Research projects	Simple, descriptive, that can be completed between 8–10 months mostly using routine programme data. Trainees receive mentorship through peer-review process to acceptance to journals.	Simple, descriptive, that can be completed within 8 months using routine programme data. Trainees receive mentorship through peer-review process to acceptance to journals.	
Data analysis	Analysis using Epidata and latterly EpiInfo.	Analysis using Stata.	Preferred using Stata for analysis to prepare trainees for advanced research in the future. Stata also provides perpetual licence (max of 5 installations), offers on-line programme help and discounted prices for organizations.
Costs	Full scholarship: tuition fees, travel expenses, full accommodation and open access publication. Other research costs covered by trainees’ home institutions.	Full scholarship: tuition fees, travel expenses and full accommodation. Each project received US$4000 for publication, conferences and research fieldwork related costs such as data collection, software, communication and travels.	Grant funding included to teach budgeting skills as well as enhance trainee experience with grant management.
Monitoring and evaluation	Participants appraise the training workshops, each participant completes milestones and submits paper to a peer-reviewed journal.	Participants complete milestones and submit paper to a peer-reviewed journal.	Participants’ appraisal of the training workshop was not conducted but is recommended for inclusion in future programmes

Notes: PIH/Rwanda: Partners In Health/Rwanda; IORT: Intermediate Operational Research Training; SORT IT: Structured Operational Research and Training Initiative; RMoH: Rwanda Ministry of Health

We broke down large competencies into smaller competencies and milestones (Table 2). ‘Developing a Research Protocol’ was comprised of four modules: developing a research question; study design and research ethics; sampling and research budgeting; and data collection and management. ‘Data Analysis’ included module five, data cleaning and management, and module six, intermediate analysis. Module seven, ‘Developing a Research Manuscript’, was on manuscript writing and publication. Each session included short lectures, break-out writing sessions with real-time mentorship and plenary sessions for group feedback, followed by a practicum period to implement skills and receive further mentorship.

**Table 2.** Course curriculum and expected milestones in PIH/Rwanda’s IORT course.

	Topics/Competencies	Milestones
**Module 1 (2 days**): Research question and protocol development	Training overview What is operational research? Research terminology Literature reviews Forming a good research question and objectives Forming a research team	Draft background, research question and objectives Review and submit summary of 5 related articles Propose research team
**Module 2 (2 days**): Study design, ethics and protocol development	Study designs Descriptive statistics (overview + Stata) Defining outcomes and exposures Research ethics	Draft methods section Invite co-investigators Develop variable table Summary of 5 related articles
**Module 3 (2 days**): Sampling, budgeting and protocol development	Intermediate statistics (overview + Stata) Sample size and sample selection Research budgeting Research clearances	Complete methods section Develop budget and study timeline Complete protocol and share with research team Summary of 5 related articles
**Module 4 (2 days**): Data collection and management	Data collection and entry Developing forms/databases Managing data in Stata Managing references	Data collection forms Ensure completion of data collection and data entry before the next module
**Module 5 (2 days)**: Data cleaning and management	Intermediate statistics (overview + Stata) Data cleaning and management	Complete data cleaning Prepare dummy tables
**Module 6 (2 days**): Intermediate analysis	Intermediate analysis Results tables and figures	Data analysis Complete results tables and figures
**Module 7 (2 days**): Paper writing and publication	Creating an outline Writing results, methods and discussion Choosing a journal Authorship, acknowledgements, abstracts, title The paper process from submission to publication	Complete manuscript Obtain co-investigator’s approval Submit to a journal

Notes: PIH/Rwanda: Partners In Health/Rwanda; IORT: Intermediate Operational Research Training

### Participant and research project selection

Eligible participants were programme or clinical professionals working in three rural districts supported by PIH/Rwanda. We targeted this category of trainees to improve problem-solving skills within clinical programmes [27], increase the use of routinely collected programme and clinical data [28], and provide a professional development opportunity to retain health workers in rural settings. With few instructors and limited operational research funding, we limited the number of training projects. However, we had applicants apply as PIH-RMoH pairs to jointly lead each project so that more individuals received training. Pairing had the added benefit of encouraging peer-to-peer learning and improving communication and collaboration among PIH/Rwanda and RMoH colleagues.

Applications included a brief research proposal describing a research question of interest and the relevance of the proposal to trainees’ work. Pairs also submitted information on previous research exposure, which most often was successful completion of the PIH/Rwanda ‘Introduction to Research’ seminar course [26] and recommendations from their supervisors. The selection committee included RMoH leadership, PIH/Rwanda leadership and training mentors. The committee considered the strength of applications, the feasibility of projects and the strategic value of projects to PIH/Rwanda and the RMoH. We selected only quantitative research proposals because course mentors were quantitative experts and to standardize the course content across teams. However, qualitative projects could be included as done in the 2017 SORT IT course [29].

### Course facilitation and mentorship

Intensive hands-on mentorship during in-person sessions and practicums was a core component of this course. The two primary mentors were a PhD-level biostatistician who had observed one iteration of a SORT IT course and an MPH-level trainer with extensive research and public health programme experience. Both mentors were already working full time in Rwanda with PIH/Rwanda. While it is not necessary for mentors to have the same credentials, it was critical that course mentors had extensive research experience with technical expertise in study design and statistical analysis to support trainees during the development of their papers. The course mentors facilitated each training session and provided concurrent mentorship to all research teams during in-person sessions and practicums for the first six modules (Table 2). In the seventh module, we matched each mentor with two to three teams to provide more focused support as trainees finalized their manuscripts. Half-way through the training, an undergraduate research fellow joined the training team as a junior mentor to coordinate training activities and provide mentorship.

Trainees received 112 contact hours during in-person training sessions (eight hours/day for two days/week for seven weeks in the course of eight months). In addition, between the sessions during practicums, the course mentors and the fellow travelled to trainees’ workplaces to provide in-person mentorship. Mentors provided an average of two hours of practicum mentorship per week per team for 25 weeks. The fellow provided four hours of practicum mentorship per week per team for 15 weeks. This totalled an estimated 110 hours of mentorship per research project during the practicum period.

### Course costs and funding

PIH/Rwanda used operational funds and innovation grants to cover training costs for all training sessions, which included meals, transport and accommodation for RMoH trainees, and amounted to $4660. PIH/Rwanda also awarded each project $4000 ($20,000 total), to cover project-specific costs such as ethics approvals, data collection, communications and co-investigator meetings. We decided to provide each project with funding so trainees could gain experience with research budgeting and grants management.

PIH/Rwanda provided in-kind support, including training facilities and equipment in Kigali, printers and transport and accommodation for PIH trainees and training staff. PIH/Rwanda also provided administrative and logistical support. In addition, training staff offered their time in-kind. Finally, participants’ time was provided in-kind by their respective employers. We offered no per diems or salary top-ups to trainees.

The course received technical and financial support from The Global Health Delivery Partnership, which includes PIH, Harvard Medical School (HMS) Department of Global Health and Social Medicine and Brigham and Women’s Hospital Division of Global Health Equity. The PhD-level biostatistician who served as a course mentor was employed by the HMS Research Core and based full time in Rwanda to lead research capacity building activities and provided technical feedback on training implementation and research projects. The HMS Research Core also funded the undergraduate research fellow.

## Results from the first Intermediate Operational Research Training

From a pool of 12 research proposals, with 24 applicants, the selection committee chose five proposals (Table 3). For the five selected projects, nine trainees completed the course. (One individual left Rwanda prior to the start of the training.) Of the trainees, only one was female. Six of the trainees were clinical staff (pharmacists and medical doctors) and the other three were programme staff. One team assessed clinician adherence to renal function monitoring guidelines for adult HIV patients [30]. Two teams addressed pharmacy issues: one evaluated prescription patterns at a district hospital outpatient clinic [31], and the other described essential medicine stock-outs at health centres [32]. Another assessed dental caries management [33], and the last team assessed using a device to manage respiratory distress in neonatal units [34].

**Table 3.** Research projects completed during PIH/Rwanda’s IORT course.

Title of research project	Author credentials	Main findings	Implication for policy and practice	Publication status
Adherence to renal function monitoring in HIV-infected patients on tenofovir-based antiretroviral therapy in rural Rwanda	Medical doctor†; previous research experience but never led or published a paper	At baseline, 50.8% of all patients had their creatinine tests ordered and 94.8% of these received a result. During the subsequent 1-, 3- and 6-month visits, between 2.3% to 9.3% of patients had their creatinine monitored.	In the immediate future, automated testing reminders generated from electronic records can help clinicians adhere to the renal function monitoring guidelines. However, guidelines should be reviewed to assess feasibility in this context and identifying safer ART therapies are recommended.	Uwamungu et al. 2016 [30] African Journal of AIDS and HIV Research
Assessing prescribing patterns of essential medicines in three rural district hospitals in Rwanda	Pharmacists; no previous research experience	Percentage of encounters with an antibiotic prescribed (37.2%) was above WHO target, while the percentage of encounters with an injection prescribed (7.2%), percentage of medicines prescribed in generic names (75.0%) or from the National Essential Medicines List (70.5%) were below WHO targets.	Monitoring and evaluation of prescribing practices should be incorporated into the national strategy as part of regular clinical audits to address prescribing behaviours.	Ntirenganya et al. 2015 [31] International Journal of Pharmacy
Assessment of essential medicine stock-outs in health centres in Burera District in Northern Rwanda	Pharmacist and social support manager; no previous research experience	73% of health facilities faced a challenge of medium to high levels of stock-outs.	Flexibility in national tender procedures to mitigate the likelihood of essential medicine stock-outs in the event of challenges in the public drug procurement system.	Nditunze et al. 2015 [32] Rwanda Journal of Medicine and Health Sciences
Dental caries management at a rural district hospital northern Rwanda: a neglected disease	M&E and PBF programme managers; no previous research experience	97.6% of the patients had their tooth extracted. In addition to dental caries, 74.9% of the patients had chronic pulpitis.	Prioritize caries prevention and care using community-based interventions and introduce advanced training, equipment and materials for dental caries management.	Mukashyaka et al. 2015 [33] Public Health Action
Bubble CPAP to support preterm infants in rural Rwanda: a retrospective cohort study	Medical doctors; Previous research experience but never led or published a paper	Of bubble CPAP-eligible infants, 59.0% were correctly identified by clinicians and 51.8% were correctly initiated on bubble CPAP.	Mentorship and refresher trainings may improve guideline adherence, particularly given high rates of staff turnover. Future qualitative and prospective research is needed to determine challenges encountered by clinicians in using bubble CPAP.	Nahimana et al. 2015 [34] BMC Pediatrics

Notes: PIH/Rwanda: Partners In Health/Rwanda; IORT: Intermediate Operational Research Training; M&E: Monitoring and Evaluation; PBF: Performance Based Financing; CPAP: Continuous Positive Airway Pathway; WHO: World Health Organization; ART: Anti-retroviral Therapy; †The second member of the team left Rwanda after selection but before the start of training, therefore this individual completed his training on his own.

All the trainees completed the milestones and wrote manuscripts. As of May 2016, all five papers had been published [30–34] (Table 3). All trainees attended every training session with occasional but rare late attendance owing to competing clinical duties. Trainees led all aspects of their research including collaborating with co-investigators on their protocol, analysis and manuscript development, manuscript submission to a peer-reviewed journal, and responding to peer review. Three teams presented their projects at local conferences and workshops. Two participants changed employer within Rwanda during the course but continued with the course and completed their milestones. Two trainees are currently pursuing research-related Masters programmes, and three have been involved in other research projects after completing the training.

## Lessons from the first Intermediate Operational Research Training

### Foundations for success

The success of the first IORT depended on national guidelines that prioritized research capacity building [22–24] and institutional support for research capacity building from PIH/Rwanda and the RMoH (Table 4). Both RMoH and PIH/Rwanda employees received time from their clinical and programme duties to attend the training and complete training milestones. PIH/Rwanda also created a research-enabling environment, implementing an ‘Introduction to Research Course’ [26] and providing budgets and staff to support research administration and training implementation. Through partnerships, the course received financial and technical support that covered the time for most of the training staff. We are in the process of conducting a systematic training costing study, accounting for in-kind costs, to estimate actual training expenses that can support other organizations planning to implement similar training.

**Table 4.** Foundations for success.

**National support**
Availability of research guidelines mandating research capacity building for Rwandans in all research activities
In response to guidelines, district hospitals support health workers to participate in research activities
**Institutional support**
Provision of time for research training and completion of milestones
Availing staff to implement and coordinate course activities
Availing budgets for each course research project
Creating research enabling environment through ‘Introduction to Research Course’ to cultivate research interest and graduate research training scholarships
**Building on successful programmes and adapting for context-specific challenges**
Identifying successful SORT IT, observing SORT IT and preserving its core elements such as output-oriented models, learning-by-doing methodology, intensive hands-on mentorship
Stepwise course implementation in setting with nascent research culture Pairing trainees for team-based project implementation in a setting with limited mentorship and funding resources
**Partnerships**
Technical feedback on training implementation from Harvard Medical School Research Core
Support with hiring course mentors from Harvard Medical School Research Core

Note: SORT IT: Structured Operational Research Training Initiative

Further, building on the successful SORT IT model saved time and resources in the planning phase, and the course adaptations ensured the training addressed contextual challenges [20]. Finally, trainee commitment, shown by training attendance and completion of milestones, sometimes even after changing employers, was key to the success of IORT. However, monitoring and evaluation of the course was limited. The initial plan was to evaluate the training at the end when papers were published. The publication dates ranged from 10 months to 1.5 years, making the evaluation timeline ineffective. Therefore, the monitoring and evaluation of this course focused only on data routinely available such as the completion of milestones, submission to and acceptance of a paper at a peer-reviewed journal and students’ formal engagement with subsequent research. We did not systematically capture participants’ assessments of course strengths and weakness but note that for future offerings more formal monitoring and evaluation activities throughout the in-person training and practicum sessions, including training evaluations to report participants’ experiences, would improve course delivery.

### Challenges for the IORT course

We navigated several challenges during the implementation of the IORT course. The first was a delay in approving research protocols (Table 5). While the original eight-month schedule allowed two months for this process, the approval process – including technical review from PIH/Rwanda, technical review from the National Health Research Committee, ethical review from the Rwanda National Ethics Committee and registration by the RMoH – took eight months, leaving a long gap between Sessions Three and Four. Other research and research training programmes attribute delays in ethics review in sub-Saharan Africa to high workload, limited capacity and inadequate resources for ethics review committees [20,35,36]. Further, the costs for ethical review in Rwanda consumed approximately 35% of the research project funds. Nesting IORT training projects into research projects that already have approvals could remove these delays and costs, but this would decrease trainee ownership of the project and the opportunity to address novel and field-driven research questions.

**Table 5.** Challenges and solutions for PIH/Rwanda’s IORT course.

Challenges	Solutions and their advantages	New challenges
**Research approval process** Long gap between training modules to obtain ethical review approvals High costs for ethical review	Implement projects that have active ethical approvals eliminating time budget for ethical review Review costs covered by the pre-approved protocols releasing budget for other training activities	Might decrease trainee project ownership when research topics are not trainee’s research interests Reduces chances for addressing novel field-driven research questions
**Mentorship** Few mentors in-country, thus few training slots Lack of funding to hire in-country or external mentors Significant hours per project investment per mentor	Train in pairs per project to increase training slots. Also encourages peer learning; strengthens institution partner relationships, and, in case of turnover, one trainee is available to complete project Increase number of junior mentors	Balance of workload between research team Negotiation of first or co-first authorship between trainee pair
**Article access and publication** Difficulty accessing articles in subscription/closed access journals High publication fees for open access publication	Identify journals that waive publication fees for low- and middle-income country authors Commit to publishing open access	Narrows the available journals for publication and decreases ease of publishing

Notes: PIH/Rwanda: Partners In Health/Rwanda; IORT: Intermediate Operational Research Training

Secondly, due to a limited mentor pool available in Rwanda and lack of funding to hire additional mentors, the few IORT mentors had a high workload. Despite limiting training slots to five research projects and hiring a research fellow to coordinate training and provide junior mentorship, we estimated 250 hours of mentorship from each senior mentor during the practicum periods in addition to 112 training hours and time taken to develop course materials and support trainees through publication. Availability of mentorship resources is an ongoing challenge for similar training [11,37,38]. Engaging more junior mentors could lower the mentorship burden on course mentors and grow a future mentorship pool. However, a long-term funding mechanism is needed to ensure the availability of course mentors and to continue and expand the IORT course to meet broader national research capacity goals. In addition, creating and strengthening a national IORT alumni network would facilitate continued participation in national research goals as well as nurture future leadership for similar programmes.

Thirdly, our trainees faced difficulties in accessing peer-reviewed published literature and in publishing their projects open access. While PIH/Rwanda is part of the World Health Organization’s Hinari Programme that facilitates access to biomedical and health literature for LMICs [39], our trainees often came across relevant literature that were closed access. Reliance on course mentors with other institutional privileges to access such articles was a short-term solution, and IORT is committed to publishing open access to facilitate local research consumption as well as reduce similar challenges for other LMICs researchers. However, open access fees were as high as $3000 in some journals, which was 75% of the total budget for each IORT research project. Recommendations for reducing publication fees [40] or making health research freely accessible to the user [41] have been made before. We encourage public health and global health research journals to have operational research sections that are open access, as the Journal of AIDS and The Lancet through the Lancet Global Health have done [42,43]. In addition, we recommend journals waive fees when first authors are from LICs, as done by PLoS and BioMed Central journals [44,45].

Finally, only one female applied to and was selected for the course. Female participation in research and research training has been a challenge in our other training activities [26] and in research capacity building generally [9]. In our course, requiring applicants to apply in pairs might have further isolated women, with male applicants pairing based on existing work relationships and perceived ease of scheduling research time outside work hours [46]. Gender inequity in research training programmes fosters gender biases in research prioritization and implementation and fails to nurture female role models in research leadership. As noted by others, similar courses should secure slots for female trainees and arrange long-term mentorship for female trainees who show talent in research [46,47].

## Conclusions

Health research systems in LMICs need adequate numbers of individuals with skills to carry national research agendas forward [38,48–50]. This IORT course included five research projects and nine trainees to ensure trainees received adequate mentorship to implement their research projects. With this combined didactic training and intensive mentorship, IORT was an effective way to cultivate Rwandan research talent to support and lead research, support the rural district-based government and NGO staff in gaining research exposure, and harness resources to ask and answer critical research questions on the successes or ongoing gaps within health programmes. While national guidelines and policies may recommend or mandate capacity building in research projects and national author involvement, focused efforts are needed to support the vision of increasing researchers and useful research outputs for policy in Rwanda and other African countries. NGOs implementing research training programmes should build on the strengths of successful research training programmes while adapting some elements to address challenges unique to each research setting [20]. IORT course welcomes the opportunity to share materials and lessons learned with others interested in conducting this type of course.

## Paper context

Recent years have seen increased calls for national research capacity building in low-income countries, with little demonstrated success. Partners In Health/Rwanda (PIH/Rwanda) adapted the WHO/TDR Structured Operational Research and Training Initiative (SORT IT) training model and reports successful course implementation and outcomes. This paper highlights considerations for other countries to implement national operational research training programmes, including ways to mitigate common challenges to successful research capacity building efforts.
